# Supplementary material for: Phylodynamic Analysis Suggests That Deer Species May Be a True Reservoir for Hepatitis E Virus Genotypes 3 and 4
Source: Microorganisms. 2023 Feb 1;11(2):375. doi: 10.3390/microorganisms11020375 (PMC9967072; doi:10.3390/microorganisms11020375)
Supplement: Supplementary file 1 [file microorganisms-11-00375-s001.zip › microorganisms-2129236-supplementary/Supplement.pdf]

Supplementary Table S1. Models used for Bayesian analysis of different HEV sequence datasets.

	Sequence subset 1		Sequence subset 2		Sequence subset 3		Sequence subset 4	
model	"-lnL"	AIC	"-lnL"	AIC	"-lnL"	AIC	"-lnL"	AIC
JC	16765,07	34554,15	14202,52	29753,05	20869,0388	43310,0776	2232,2608	4636,5216
JC+I	15314,93	31655,86	13094,88	27539,76	19266,7387	40107,4775	1977,4460	4128,8921
JC+G	14101,53	29229,06	12197,20	25744,40	17646,3899	36866,7798	1919,4280	4012,8561
JC+I+G	14091,41	29210,81	12169,91	25691,81	17647,2055	36870,4110	1926,0172	4028,0343
F81	16390,68	33811,36	14030,37	29414,73	20193,9578	41965,9155	2221,8965	4621,7930
F81+I	14950,57	30933,14	12926,70	27209,40	18599,7378	38779,4756	1969,7905	4119,5810
F81+G	13535,97	28103,94	11906,13	25168,26	16691,8476	34963,6951	1899,8483	3979,6967
F81+I+G	13490,01	28014,02	11888,38	25134,76	16620,3300	34822,6600	1906,8889	3995,7777
K80	15082,80	31191,60	12749,13	26848,26	18881,0136	39336,0272	2114,4796	4402,9591
K80+I	13591,15	28210,31	11576,41	24504,82	17256,1051	36088,2102	1832,4891	3840,9783
K80+G	12208,36	25444,71	10519,69	22391,38	15464,6154	32505,2308	1765,5960	3707,1920
K80+I+G	12194,26	25418,53	10521,11	22396,23	15488,9741	32555,9482	1769,6402	3717,2804
HKY	14869,13	30770,26	12604,55	26565,09	18375,5486	38331,0972	2093,0428	4366,0856
HKY+I	13379,05	27792,10	11471,60	24301,20	16816,3867	35214,7734	1823,3928	3828,7857
HKY+G	12088,10	25210,20	10445,87	22249,73	15248,0517	32078,1033	1756,3885	3696,7769
HKY+I+G	12087,52	25211,04	10448,03	22256,07	15267,2246	32118,4492	1763,9472	3711,8944
SYM	14776,36	30586,71	12576,19	26510,37	18285,4280	38152,8561	2083,3756	4348,7511
SYM+I	13330,25	27696,51	11430,84	24221,68	16733,1503	35050,3005	1814,1634	3812,3268
SYM+G	12078,40	25192,81	10435,50	22231,01	15267,4100	32118,8200	1753,8094	3699,6188
SYM+I+G	**12018,77**	**25079,53**	10450,22	22262,44	15278,6300	32143,2600	1760,4260	3706,8520
GTR	14755,96	30551,93	12552,06	26468,12	18194,5989	37977,1977	2076,5844	4341,1688
GTR+I	13308,08	27658,16	11424,36	24214,71	16695,0191	34980,0382	1817,3856	3824,7713
GTR+G	12083,10	25204,20	10411,04	22190,07	**15222,3188**	**32034,6376**	1755,1423	3700,2845
GTR+I+G	12024,79	25093,58	**10399,50**	**22165,01**	15223,3383	32038,6766	**1755,8540**	**3693,7080**

"-lnL" - Negative log-likelihood
AIC - Akaike information criterion
The smallest values of the criterion are highlighted in red bold

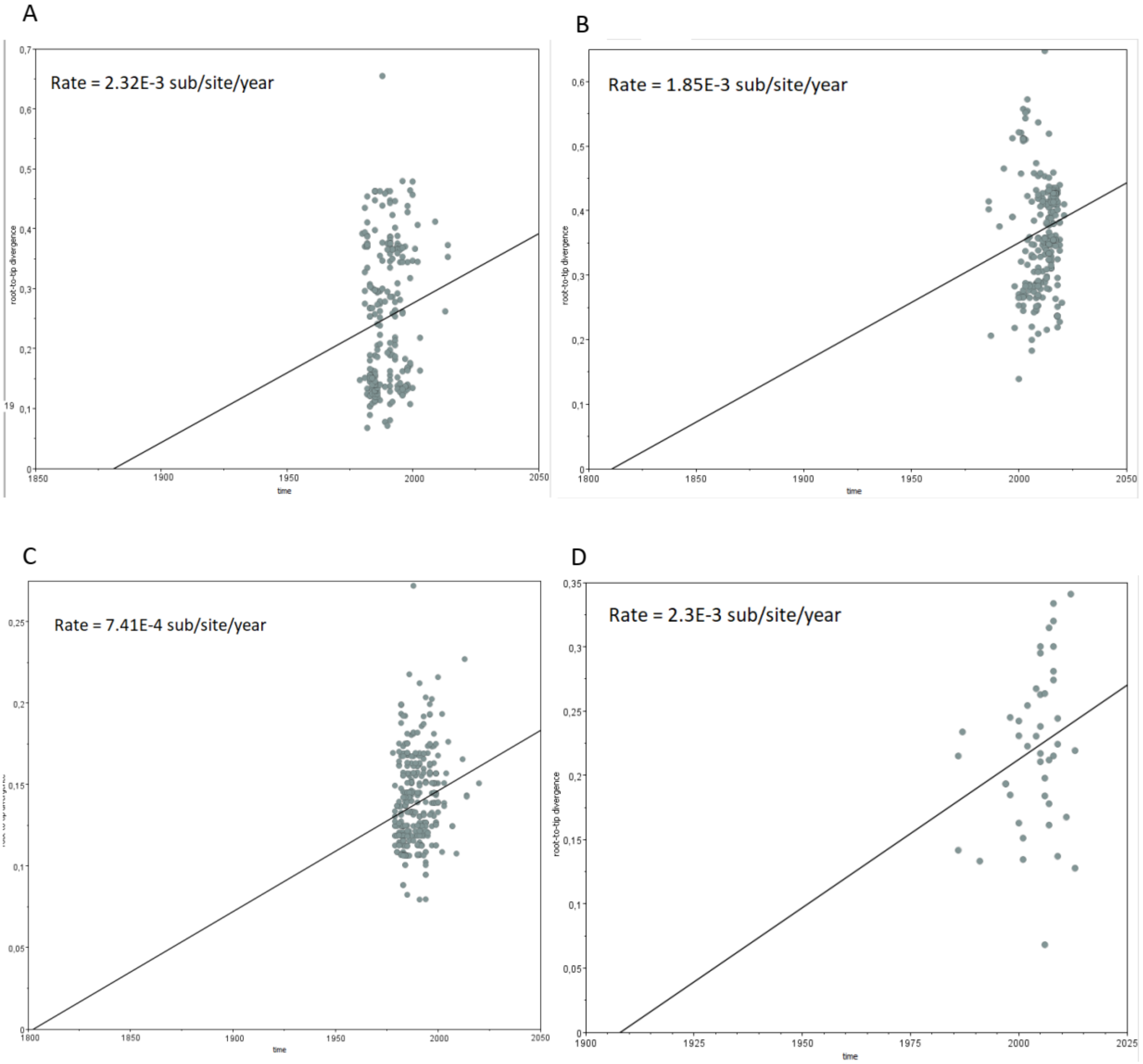

Supplementary Figure S1. Temporal signal linear regression graphs for four HEV sequence subsets: ORF1 sequences, nt positions 125 – 450 (A); ORF1 sequences, nt positions 4071 – 4350 (B); ORF2 sequences, nt positions 5762 – 6105 (C); ORF2 sequences, nt positions 6109 - 6256. Nt positions are given by reference sequence M73218.
